# Plasminogen activator inhibitor 1 and gestational diabetes: the causal relationship

**DOI:** 10.1186/s13098-022-00900-2

**Published:** 2022-09-08

**Authors:** Gaber El-Saber Batiha, Hayder M. Al-kuraishy, Thabat J. Al-Maiahy, Ali K. Al-Buhadily, Hebatallah M. Saad, Ali I. Al-Gareeb, Jesus Simal-Gandara

**Affiliations:** 1 Department of Pharmacology and Therapeutics, Faculty of Veterinary Medicine, Damanhour University, Damanhour, 22511 Egypt; 2 Department of Pharmacology, Toxicology and Medicine, College of Medicine, Al-Mustansiriyah University, P.O. Box 14132, Baghdad, Iraq; 3 Department of Gynecology and Obstetrics, College of Medicine, Al-Mustansiriyah University, P.O. Box 14132, Baghdad, Iraq; 4 Department of Clinical Pharmacology, Medicine and Therapeutic, Medical Faculty, College of Medicine, Al Mustansiriyah University, P.O. Box 14132, Baghdad, Iraq; 5 Department of Pathology, Faculty of Veterinary Medicine, Matrouh University, Marsa Matruh, 51744 Egypt; 6 Department of Pharmacology, Toxicology and Medicine, College of Medicine Al-Mustansiriya University, P.O. Box 14132, Baghdad, Iraq; 7 Nutrition and Bromatology Group, Department of Analytical Chemistry and Food Science, Faculty of Science, Universidade de Vigo, E-32004 Ourense, Spain

**Keywords:** Plasminogen activator inhibitor 1, Gestational diabetes, Hypofibrinolysis, Thrombotic complications

## Abstract

Plasminogen activator inhibitor 1 (PAI-1) also known as serpin E1 or endothelial plasminogen activator inhibitor, is produced from endothelial cells and adipose tissue. PAI-1 inhibits tissue plasminogen activator (tPA) and urokinase (uPA) preventing activation of plasminogen and fibrinolysis. Gestational diabetes mellitus (GDM) is defined as glucose intolerance and hyperglycemia during pregnancy. The underlying mechanism of GDM is due to the reduction of insulin secretion or the development of insulin resistance (IR). Normal PAI-1 is a crucial mediator for maintaining pregnancy, though aberrantly high PAI-1 promotes inflammation and thrombosis with increased risk of pregnancy loss. Increasing PAI-1 level had been shown to be an early feature of cardio-metabolic derangement in women with GDM. As well, GDM is regarded as an independent predictor for increasing PAI-1 levels compared to normal pregnancy. Taken together, GDM seems to be the causal factor in the increase of PAI-1 via induction of IR, hyperglycemia and hypertriglyceridemia. In conclusion, GDM triggers expression and release of PAI-1 which linked with GDM severity due to exaggerated pro-inflammatory and inflammatory cytokines with the development of IR. High PAI-1 levels in GDM may induce hypofibrinolysis and thrombotic complications.

## Introduction

Plasminogen activator inhibitor 1 (PAI-1) also known as serpin E1 or endothelial plasminogen activator inhibitor which is produced from endothelial cells and adipose tissue [1]. PAI-1 is also produced from megakaryocytes, platelets, monocytes, macrophages, cardiomyocytes, adipocytes, vascular smooth muscles, endometrium, mesothelium, and hepatocytes [2]. More than 90% of synthesized PAI-1 is stored in the platelets granules, the rest remains circulated or deposited in the subendothelial matrix [3]. These findings suggest that the plasma level of PAI-1 does not reflect its concentration in the formed thrombus, as it is released from platelets when the coagulation cascade is activated.

PAI-1 inhibits tissue plasminogen activator (tPA) and urokinase (uPA), preventing activation of plasminogen and fibrinolysis [4]. Besides, PAI-2 is released from the placenta, and represents the main form of PAI in pregnancy [1]. Of note, protease nexin acts as an inhibitor of urokinase and of PAI. Moreover, PAI-1 inhibits the activity of matrix metalloproteinase (MMPs) [1, 4].

Congenital deficiency of PAI-1 has been shown to cause bleeding tendency due to uncontrolled fibrinolysis. Though, a high PAI-1 level increases the risk of atherosclerosis, thrombosis and cardio-metabolic complications as in obesity, diabetes mellitus (DM), metabolic syndrome and cancer [4–6]. A high level of angiotensin II (AngII) induces the expression of PAI-1 causing the development of cardio-metabolic complications [7].

**Fig. 1:**
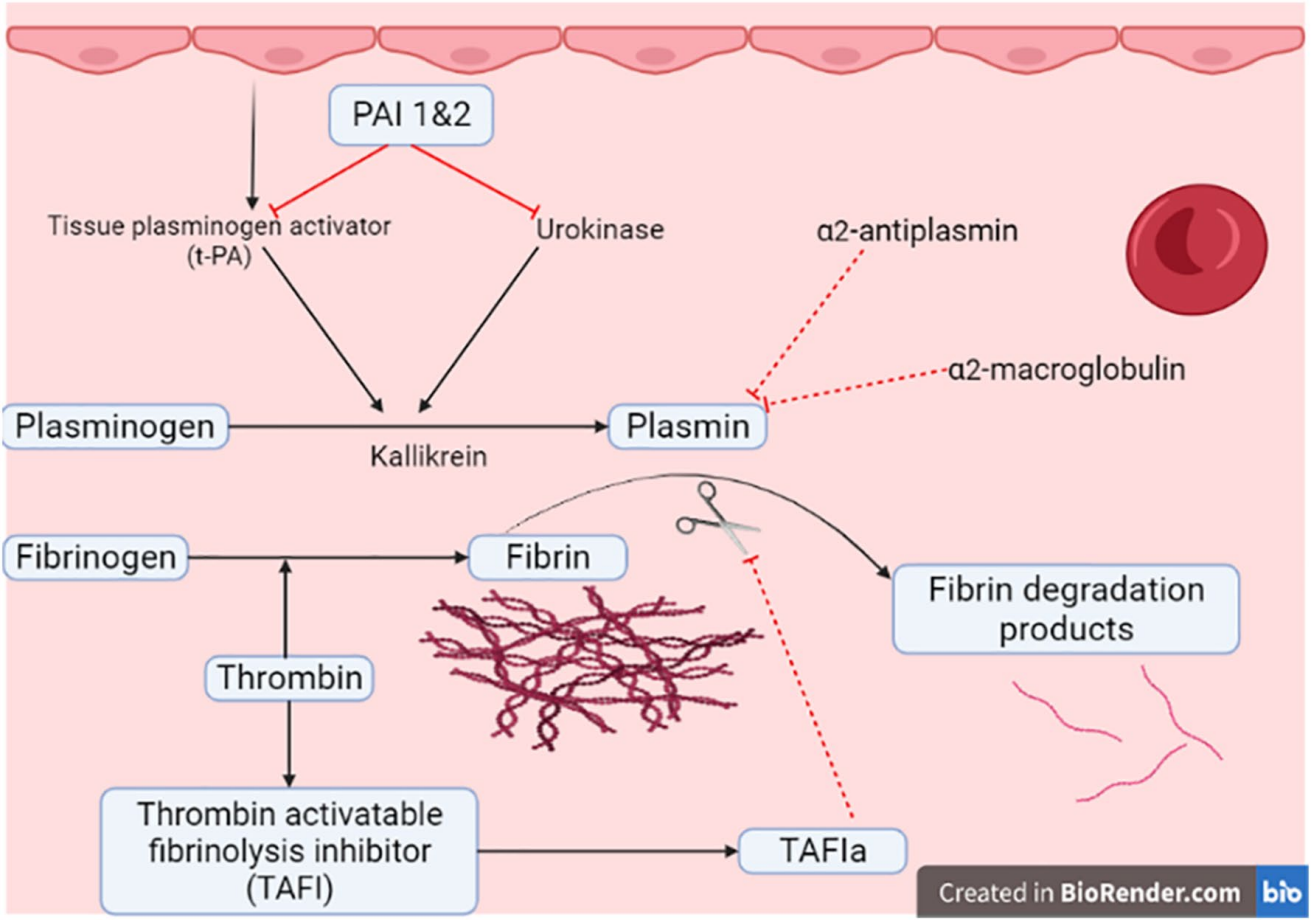
Plasminogen activator inhibitor 1 (PAI-1) and fibrinolytic pathway

PAI-1 and PAI-2 inhibit both tPA and uPA which provoke the conversion of plasminogen to plasmin. As well, kallikrein and factor XI block this pathway. In addition, α2-antiplasmin and α2-macroglobulin directly block the effect of plasmin [1] (Fig. 1)

On the other hand, gestational DM (GDM) is defining as glucose intolerance and hyperglycemia during pregnancy [8, 9]. The prevalence of GDM is about 14.3% and some studies reported that GDM affects about 3–9% of total pregnancies depending on certain populations [8]. It affects 1% under the age of 20 years whereas it affects more up to 13% of pregnant women over the age of 44 years [10]. GDM is more common among certain ethnic groups including Asians, Australian, American Indians, and Pacific Islanders. It has been reported that GDM was resolved in 90% of cases after delivery [10]. Though, pregnant women with GDM are at high risk for the development of type 2 DM (T2DM), preeclampsia and depression [8, 11, 12]. Neonates from mothers with GDM may develop hypoglycemia, severe jaundice, and large body weight [8]. Besides, long-term complications of children born from mothers with GDM are at risk for the development of overweight and T2DM [8, 10].

The underlying mechanism of GDM is due to the reduction of insulin secretion or the development of insulin resistance (IR) [13]. The risk factors for the development of GDM are overweight, obesity, previous GDM, positive family history of T2DM, and polycystic ovarian syndrome [13]. GDM is prevented by weight reduction and regular exercise. GDM is often managed by regular exercise, controlled diabetic diet, and metformin pharmacotherapy [14, 15]. GDM is classified into type A (gestational diabetes) and type B (pre-gestational diabetes). Type A1 has only abnormal glucose tolerance test with normal fasting and postprandial blood glucose. Type A2 has abnormal glucose tolerance test with high fasting and postprandial blood glucose [14].

Women with GDM are presented with polydipsia, fatigue, vomiting, recurrent urinary tract infections, and blurred vision. High placental hormones and inflammatory cytokines increase the development of IR and pancreatic cell adaptation [13, 14, 16].

**Fig. 2:**
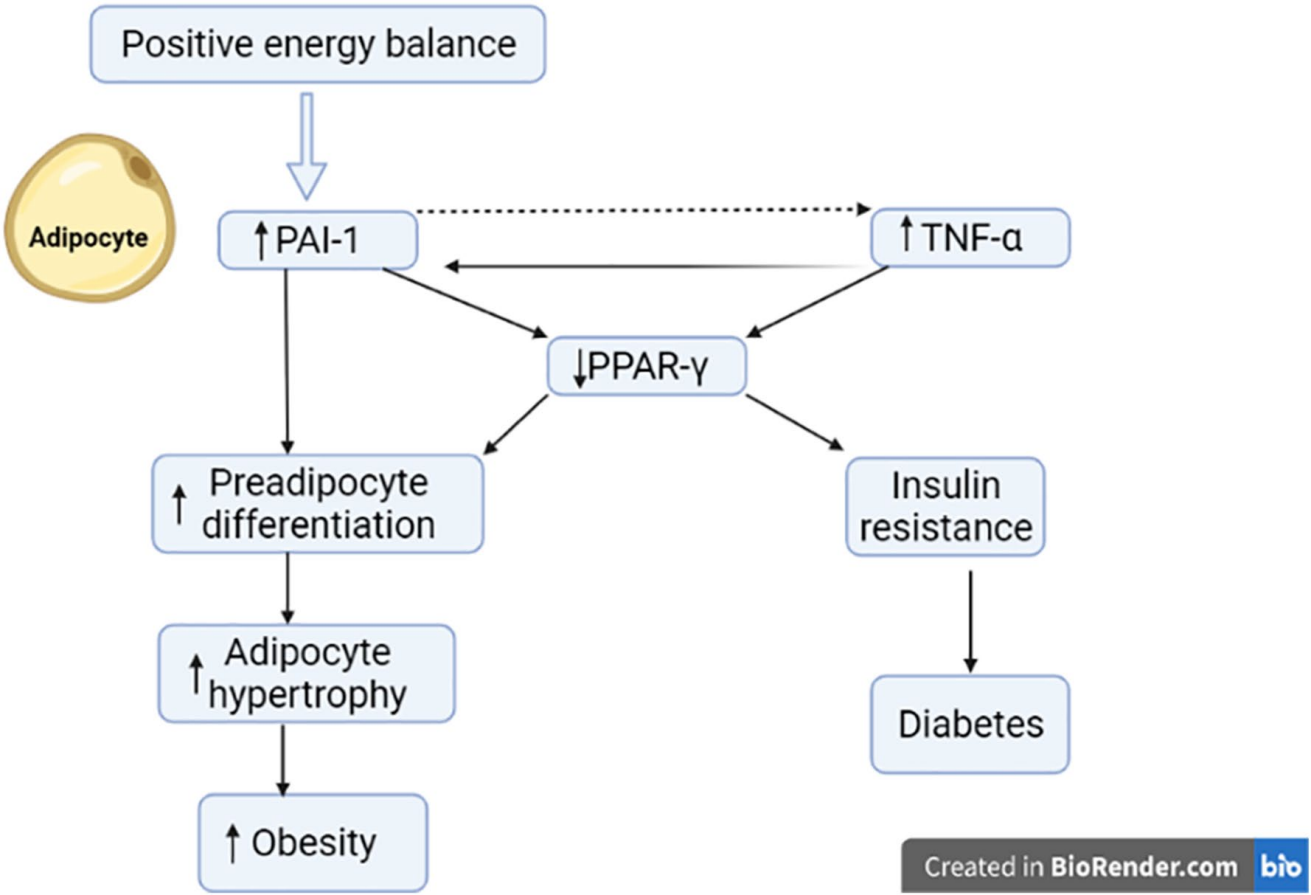
Role of plasminogen activator inhibitor 1 (PAI-1) in diabetes and obesity: Positive energy balance increase tumor necrosis factor-alpha (TNF-α) and PAI-1 leading to reduction in the expression of peroxisome proliferator activator receptor gamma (PPAR-γ) with subsequent development of insulin resistance (IR) and diabetes. Also, the reduction of PPAR-γ increases adipocyte hypertrophy and the development of obesity

Normal PAI-1is a crucial mediator for maintaining pregnancy, though aberrantly high PAI-1 promotes inflammation and thrombosis with increased risk of pregnancy loss [17]. Of note, PAI-1 is increased during T2DM and linked with the development of macrovascular complications in T2DM and obesity [18, 19] (Fig. 2). Improvement of bodyweight and IR by insulin-sensitizing agents may reduce PAI-1 and its related complications [20]. As well, polymorphism of PAI-1 is linked with the development of IR and GDM [20].

Thus, the objective of the present study was to reveal the connection between PAI-1 and the pathogenesis of GDM and related complications.

## Plasminogen activator inhibitor 1 and risk of gestational diabetes

It has been shown that PAI-1 was increased in T2DM and contributes to the hypofibrinolytic state and the development of thrombotic complications by promoting intimal injury and plaque formation [18, 21, 22]. Normally, PAI-1 plasma concentration is 10–50 ng/mL which may increase up to 100 ng/mL in presence of IR, DM and obesity [23]. PAI-1 plasma concentration is peak at the morning which falls in the afternoon due to the stimulation of PAI-1 by cortisol at the morning [23]. PAI-1 is higher in males, though variation in the level of PAI-1 is related to adipose tissue distribution and ethnicity [4, 19].

Of note, increasing of PAI-1 level had been shown to be an early feature of cardio-metabolic derangement in women with GDM [19, 20]. As well, GDM is regarded as an independent predictor for increasing PAI-1 levels compared to normal pregnancy [20]. High PAI-1 is linked with a reduction in insulin sensitivity [24]. In addition, PAI-1 through its dysglycemic effect may induce the risk of coronary heart disease and other cardio-metabolic complications [25, 26]. The potential link between PAI-1 and GDM needs longitudinal studies to confirm this association. Thus, the relationship between GDM and PAI-1 need to be verified in this claim.

In GDM, there are various factors that regulate the expression of PAI-1 including hyperglycemia, hyperinsulinemia, pro-inflammatory cytokines and high AngII [27, 28]. These inflammatory mediators increase the expression of PAI-1 by adipocytes. Besides, PAI-1 induces the expression of AngII leading to endothelial dysfunction and procoagulation state [28]. In addition, IR, hyperinsulinemia, and hyperglycemia induce the expression of PAI-1 through mitogen-activated protein kinase (MAPK) [29]. Levels of active and total PAI-1 are indeed increased in response to heightened inflammatory state and increased insulin levels, nevertheless, GDM is not a causative factor of IR, but rather IR and hyperglycemia elevate the risk of GDM [28].

Interestingly, Cao et al. experimental study confirmed that nod like receptor pyrin 3 (NLRP3) inflammasome was upregulated and involved in the pathogenesis of GDM [30]. In an experimental study, administration of tPA aggravates hyperglycemia-induced stroke through upregulation of NLRP3 inflammasome, high mobility box protein 1 (HMGB-1), tumor necrosis factor-alpha (TNF-α) and nuclear factor kappa B (NF-κB) [31]. These findings proposed that PAI-1 may attenuate hyperglycemia-induced activation of tPA.

Improvement of IR by regular exercise and metformin reduces the level of PAI-1 [1, 32, 33]. It has been reported that metformin can reduce PAI-1 by enhancing insulin sensitivity. Furthermore, polymorphism of PAI-1 is linked with the development of dyslipidemia [34]. Notably, polymorphism of PAI-1 increases the risk for the development of cardiovascular complications in patients with T2DM [35]. Herein, a high PAI-1 level in GDM may counterbalance the development of cardio-metabolic complications. In contrast, a high PAI-1 level in T2DM patients with metabolic syndrome may reduce the protective adiponectin level [36]. A cross-sectional study involved 379 T2DM with metabolic syndrome illustrated that PAI-1 level was higher and adiponectin was lower compared to T2DM without metabolic syndrome [36]. As well, increases in PAI-1 levels are reported when adipocytes are stimulated by TNF-α, transforming growth factor-β, angiotensin II, glucocorticoids, insulin, hypoxia, and ROS suggesting that PAI-1 might play a role in inflammatory mechanisms while also affecting vasculature, adiposity, IR and metabolic syndrome [36, 37].

**Fig. 3:**
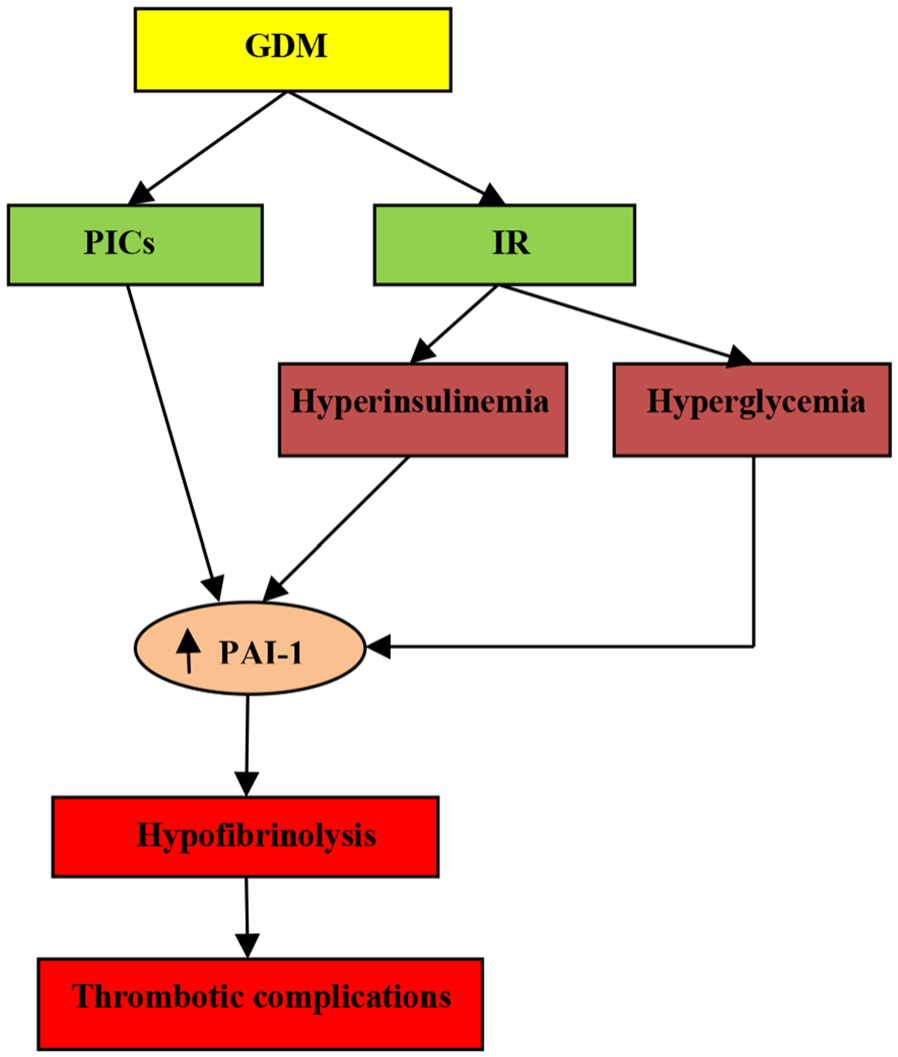
Gestational diabetes mellitus (GDM) and thrombotic complications: GDM induces insulin resistance (IR) and release of pro-inflammatory cytokines (PICs), by which leads to hyperglycemia and hyperinsulinemia. These changes induce the expression of plasminogen activator inhibitor 1 (PAI-1) with subsequent hypofibrinolysis and thrombotic complications

Taken together, GDM seems to be the causal factor in the increase of PAI-1 through induction of IR and associated hyperglycemia and hypertriglyceridemia (Fig. 3).

The present review had several limitations including paucity of clinical studies and sequential levels of PAI-1 in the different trimesters were not revealed. However, this review highlighted that PAI-1 could be a surrogate biomarker for the severity of GDM.

## Conclusions

PAI-1 level is linked with GDM severity due to exaggerated pro-inflammatory cytokines and inflammatory cytokines with the development of IR. High PAI-1 level in GDM may induce hypofibrinolysis and thrombotic complications. PAI-1 is not a potential cause of GDM, but it seems to be a consequence of metabolic derangements. Experimental, preclinical and clinical studies are warranted in this regards.

## Data Availability

Not applicable.

## Abbreviations

PAI-1: Plasminogen activator inhibitor 1
tPA: Tissue plasminogen activator
uPA: Urokinase
GDM: Gestational diabetes mellitus
IR: Insulin resistance
MMPs: Matrix metalloproteinases
DM: Diabetes mellitus
AngII: Angiotensin II
T2DM: Type 2 DM
MAPK: Mitogen activated protein kinase
NLRP3: Nod like receptor pyrin 3
HMGB-1: High mobility box protein 1
TNF-α: Tumor necrosis factor alpha
NF-κB: Nuclear factor kappa B
PPAR-γ: Peroxisome proliferator activator receptor gamma
